# A High-Pointing-Accuracy Implementation Method for an 18-m Antenna Based on a Multi-Error-Source Coupled Model

**DOI:** 10.3390/s26165201

**Published:** 2026-08-17

**Authors:** Wei Zhang, Gengxin He, Jinqing Wang, Tianzhi Yu, Fan Wang, Hailing Zhou, Rong Luo

**Affiliations:** 1 The 39th Research Institute of China Electronics Technology Group Corporation, Xi’an 710076, China; zhw0917@126.com (W.Z.); 13519199263@139.com (G.H.); jassy_yu@163.com (T.Y.); wangf_up@163.com (F.W.); zhling871229@163.com (H.Z.); luorong3456@163.com (R.L.); 2 Shaanxi Key Laboratory of Antenna and Control Technology, Xi’an 710076, China; 3 Shanghai Astronomical Observatory, Chinese Academy of Sciences, Shanghai 200030, China; 4 Key Laboratory of Radio Astronomy, Chinese Academy of Sciences, Nanjing 210023, China; 5 Shanghai Key Laboratory of Space Navigation and Positioning Techniques, Shanghai 200030, China

**Keywords:** pointing accuracy, multi-error-source coupled modeling, hierarchical compensation, TPOINT model calibration

## Abstract

**Highlights:**

**What are the main findings?**

- A multi-error-source coupled model is established, unifying geometric, gravity, wind, and dynamic errors, and revealing their coupling mechanisms (geometric-physical, control-structure, and error-synthesis coupling).
- Using RMS@95% and RSS synthesis, the total pointing error of the 18-m antenna is budgeted at 9.9 arcseconds (at 40 GHz), with wind-induced deformation (7.60 arcseconds) identified as the dominant error source.
- A three-level hierarchical compensation strategy is proposed—mechanical adjustment, TPOINT parametric calibration, and active servo suppression—with explicit mapping between physical error sources and TPOINT model parameters (e.g., IA, IE, NPAE, AN, CA, CE, GCE).

**What are the implications of the main findings?**

- The achieved 9.9-arcsecond accuracy meets the stringent ≤ 1/10 half-power beamwidth requirement for 40 GHz VLBI observations, enabling high-frequency interferometry with minimal signal attenuation and phase distortion.
- The transparent error-source–to-parameter mapping transforms calibration from black-box fitting to physical diagnostics, improving debugging efficiency and providing a systematic engineering framework for precision design and implementation of large high-frequency antennas.
- The clear identification of wind load as the primary error source highlights the need for enhanced environmental disturbance mitigation, and the hierarchical structure offers a modular pathway for future adaptive compensation under harsher conditions (higher winds or faster scanning).

**Abstract:**

High-frequency VLBI (Very Long Baseline Interferometry) observations impose stringent requirements on antenna pointing accuracy. This paper proposes a high-precision pointing implementation method that integrates a multi-error-source coupled model with hierarchical compensation. Taking an 18-m antenna as the object of study, the influence of gravity is quantified through structural-electromagnetic coupled simulation, establishing a unified model encompassing geometric, random, environmental, and dynamic errors. Using the 95% confidence bound (defined as 1.96σ for each error component) and the RSS (Root Sum of Squares) synthesis method, the budgeted system pointing error is estimated to be 8.64 arcseconds (at 40 GHz). An innovative three-level compensation system of “mechanical adjustment—model calibration—active suppression” is constructed, clarifying the mapping relationship between error sources and the TPOINT (Telescope POINT) model. To validate the proposed approach, actual radio source tracking experiments were conducted on the 18-m antenna. The experimental results demonstrate that after applying the three-level compensation strategy, the residual pointing error is reduced to approximately 7.9″ (RMS@95%), which agrees well with the theoretical budget of 8.64″. This method provides a systematic solution for the precision design and engineering implementation of high-frequency antennas, with the effectiveness confirmed through both theoretical budgeting and experimental validation.

## 1. Introduction

Very Long Baseline Interferometry (VLBI) [1,2,3,4,5,6,7], with its ultra-high resolution at the micro-arcsecond level, has become a core tool in cutting-edge fields such as gravitational wave detection and pulsar research. As observational frequencies extend into the 40 GHz and higher bands, extreme demands are placed on the pointing accuracy [8] of individual radio telescopes within VLBI networks: the pointing error must be better than 1/10 of the half-power beamwidth. For an 18-m antenna at 40 GHz, the theoretical Half-Power Beamwidth (HPBW) is approximately 0.084° (5.04 arcminutes) based on the classical approximation $\theta_{HPBM}\approx 70\lambda /D$, corresponding to a 1/10 HPBW requirement of about 30 arcseconds. The actual HPBW may vary slightly with aperture illumination efficiency and surface accuracy, and can be obtained from full-wave electromagnetic simulations (e.g., GRASP) using the deformed reflector profiles from Section 2.1. A pointing error exceeding this limit causes significant signal gain attenuation; in cases where the beam pattern is asymmetric, pointing errors may also introduce interferometric phase errors that can affect the accuracy of VLBI phase-referencing measurements.

Pointing accuracy represents a typical system engineering challenge, with error sources being complex and coupled [9,10,11,12,13,14,15,16,17]. These primarily include: (1) Systematic geometric errors, such as axis non-orthogonality and encoder zero-point offsets; (2) Structural gravity deformation varying with elevation angle [18]; (3) Random errors induced by wind disturbance and assembly tolerances; (4) Dynamic lag and nonlinear friction in servo tracking [19]. Existing research predominantly focuses on the independent analysis or final calibration of specific error types, lacking a comprehensive methodology spanning from physical mechanisms to systematic suppression. This results in distorted accuracy budgets, inefficient calibration, and insufficient synergy among various compensation measures.

This study presents a high-precision pointing realization method based on “multi-error-source coupled modeling and hierarchical compensation” for an 18-m VLBI antenna system. The approach establishes a complete technical framework covering “mechanism–modeling–budgeting–compensation.” First, a coupled model is developed to uniformly characterize multi-physics field errors and their interactions. Consistent statistical criteria and error synthesis theory (e.g., RSS (Root Sum of Squares) are then applied to reliably budget from component tolerances to system accuracy. Finally, an innovative three-level cooperative compensation system is implemented: precision mechanical adjustment forms the foundation; a parametric model (TPOINT (Telescope POINTing model)) [20] calibrates systematic errors; and active servo suppression counters random and dynamic disturbances. The physical mapping between error sources and model parameters is clarified to optimize compensation strategies and improve engineering efficiency.

## 2. Multi-Error-Source Coupled Modeling Method

The “Multi-Error-Source Coupled Modeling Method” establishes a unified framework for systematic modeling.

### 2.1. Structural-Electromagnetic Performance Coupled Modeling and Analysis

Antenna electrical performance is highly sensitive to structural deformation. Distortions of the reflector surface or displacements of the feed/sub-reflector alter the antenna’s optical path, resulting in beam deviation.

#### 2.1.1. Structural Deformation Analysis

**Figure 1:**
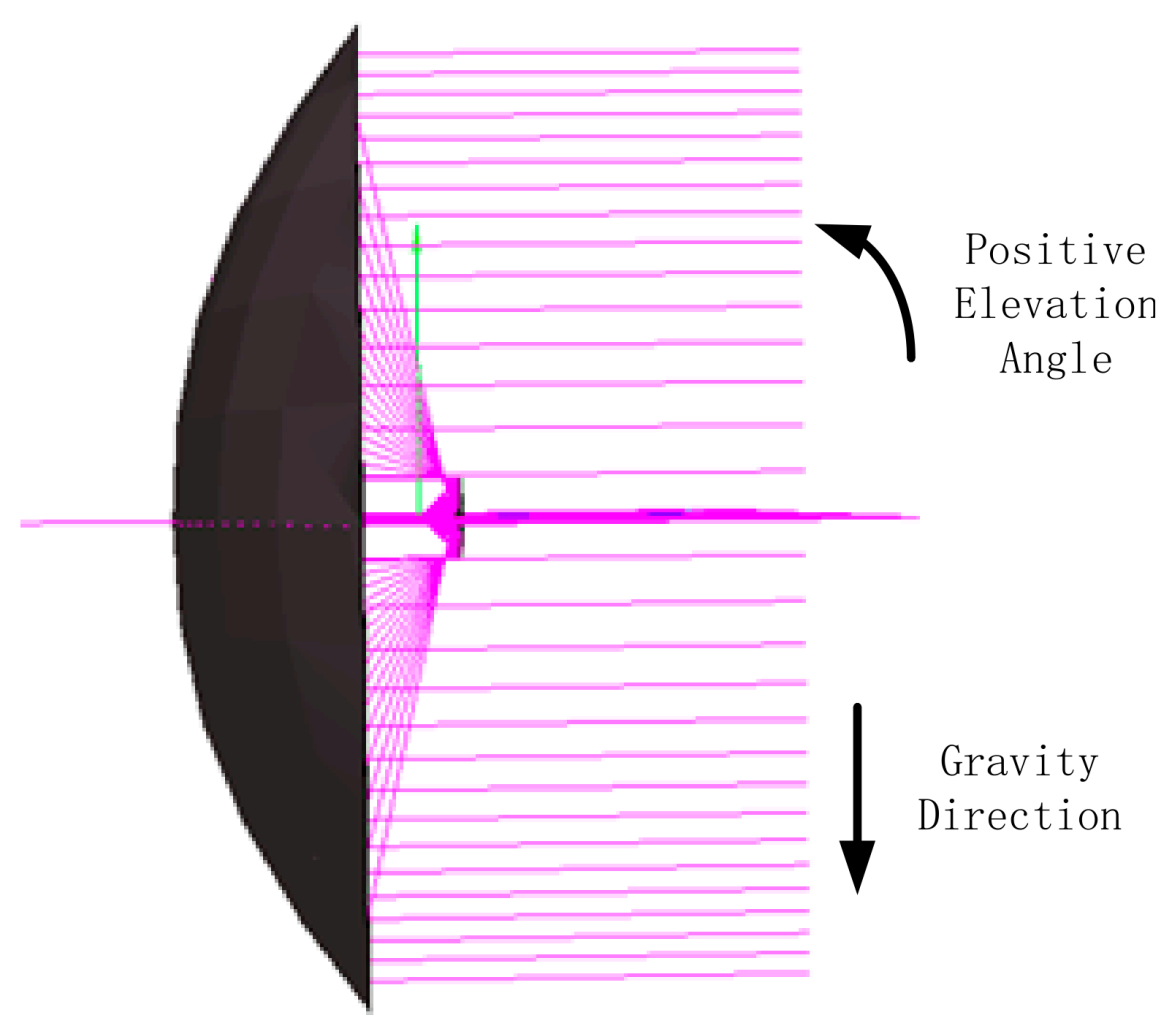
Ideal Main Reflector.

**Figure 2:**
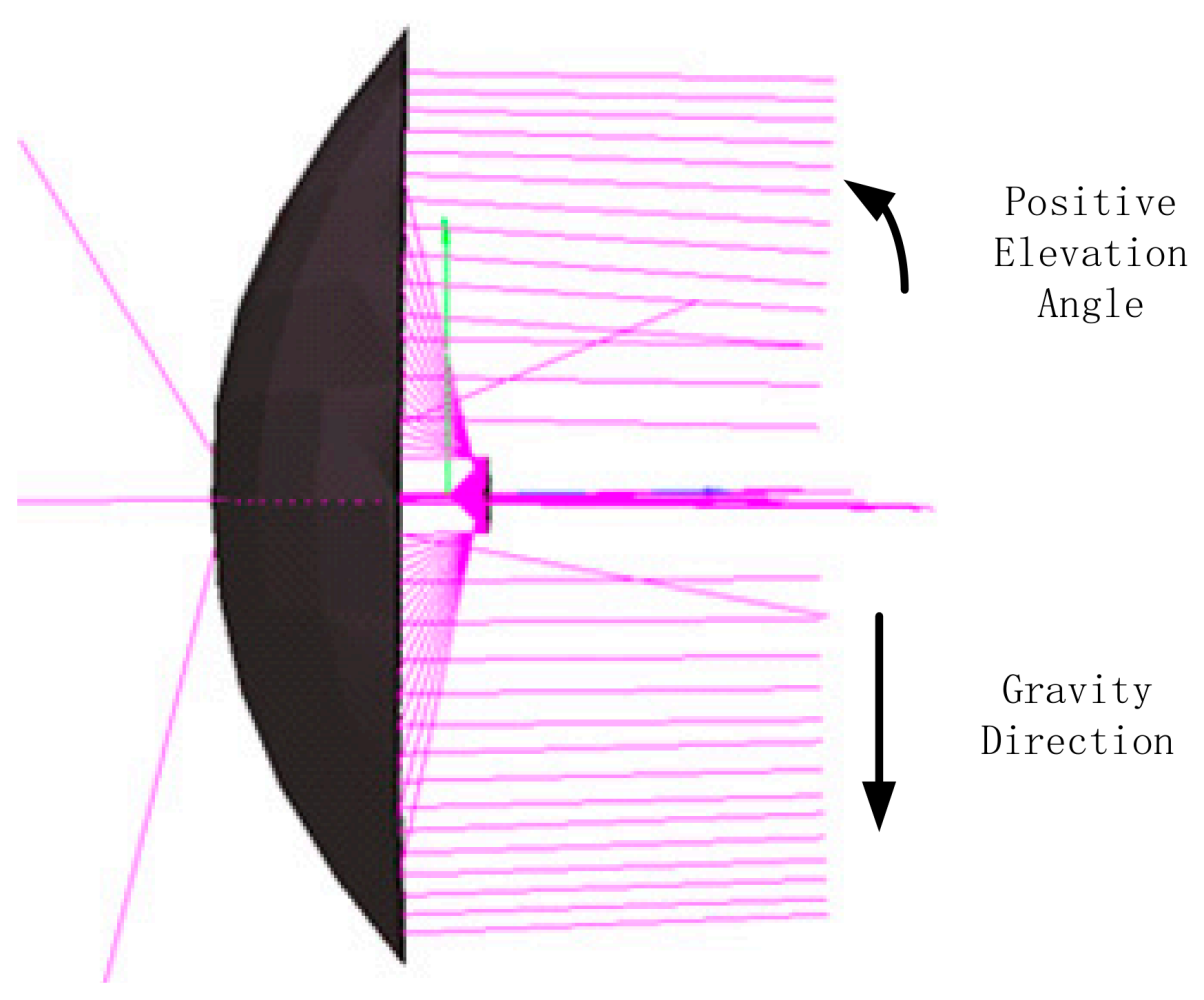
Deformed Main Reflector.

Under its own gravity, the main reflector deforms to varying degrees at different elevation angles due to limited surface accuracy, subsequently affecting the radiation pattern. The simulated profiles of the main reflector before and after deformation are shown in Figure 1 and Figure 2.

#### 2.1.2. Structural Deformation Simulation

**Figure 3:**
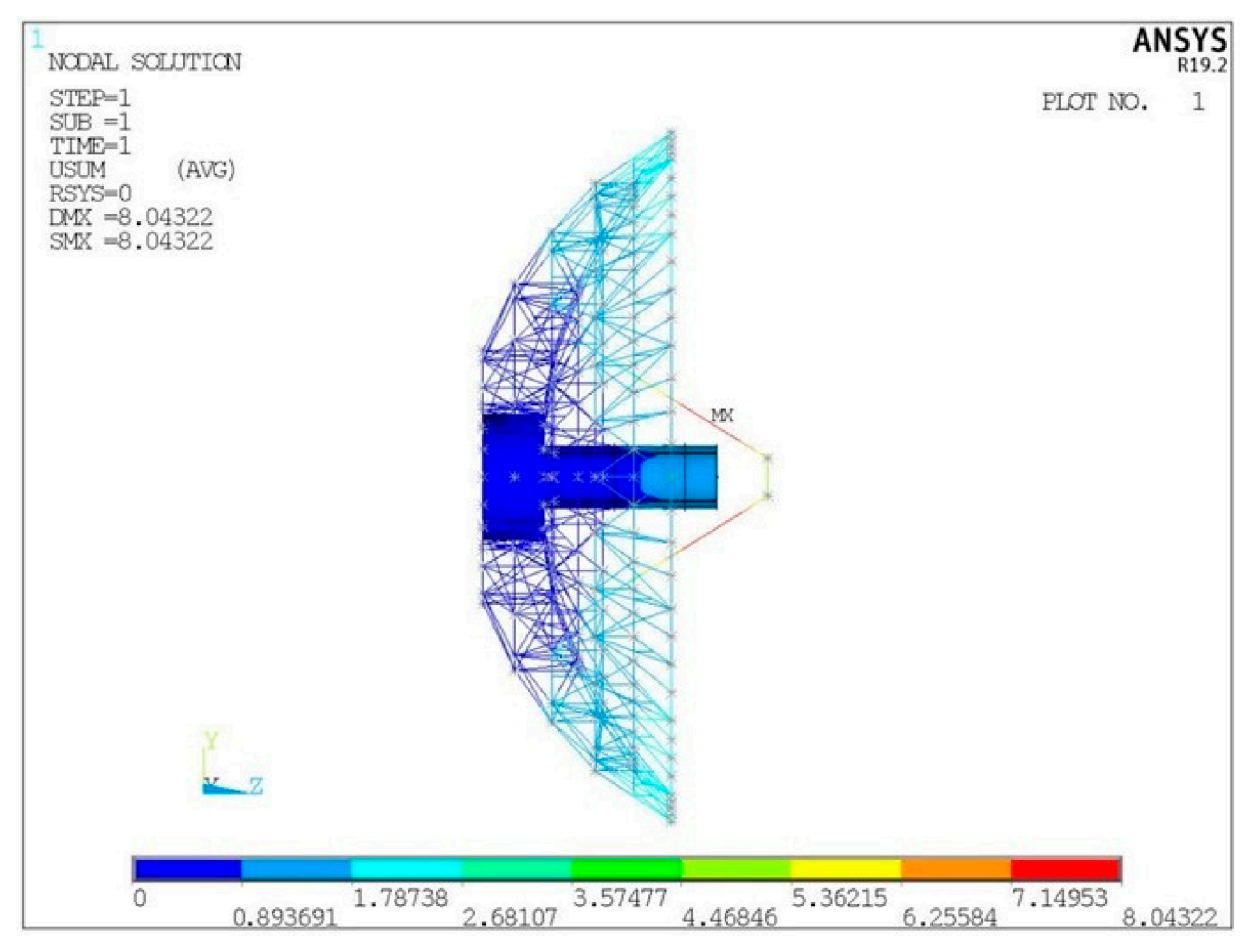
Antenna Simulation Model(mm).

**Figure 4:**
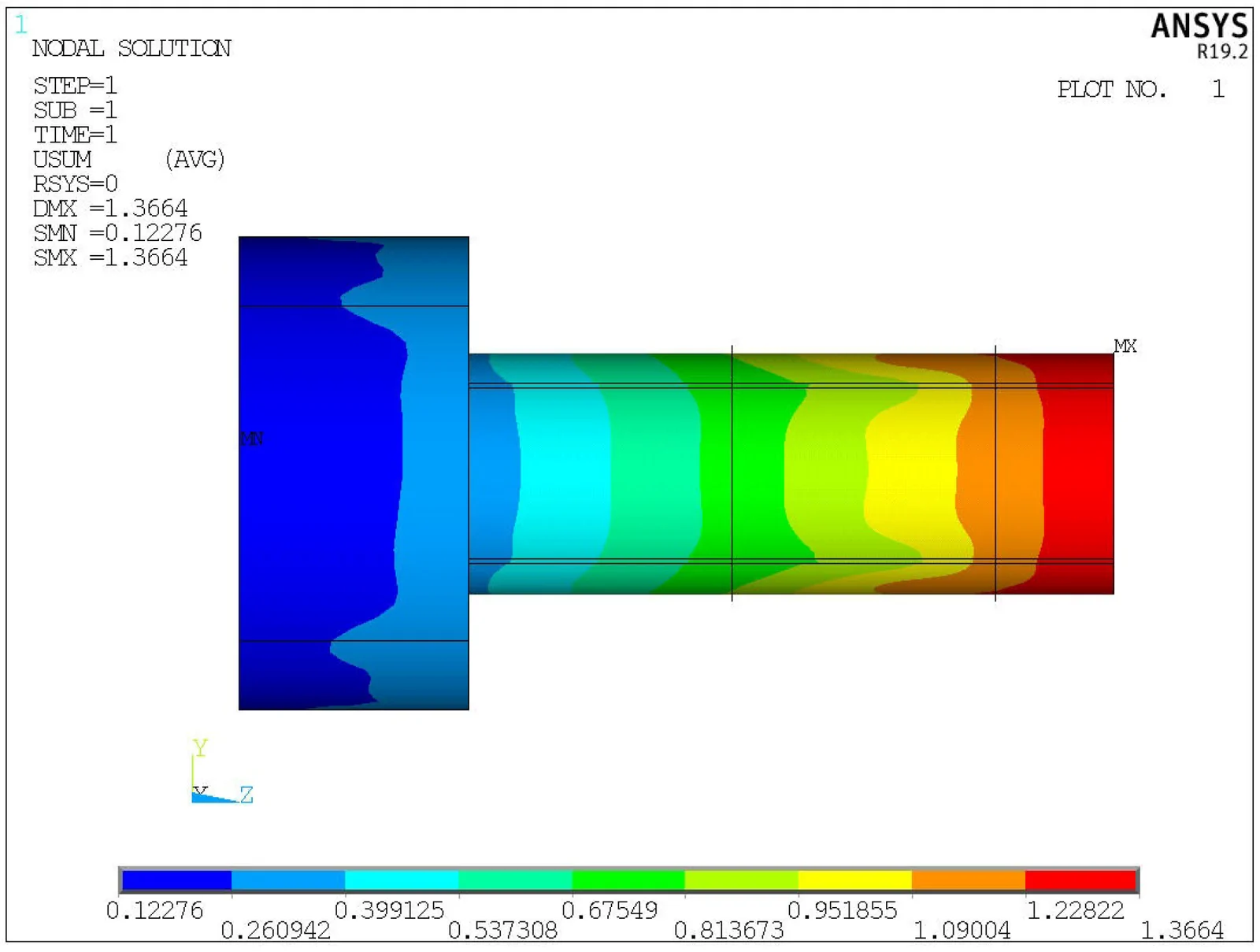
Displacement of the Feed Support Legs(mm).

To quantify the structural deformation under the coupled effects of gravity and wind load, the simulation in this section sets the ambient wind speed to 4 m/s (consistent with the boundary condition for wind disturbance in the error budget). The results shown in Figure 3 and Figure 4, and Table 1 are all obtained under this combined load condition, aiming to reveal the patterns of gravitational deformation and its specific behavior in the simultaneous presence of wind load.

#### 2.1.3. Antenna Electrical Performance Evaluation and Coupled Effect Revelation

Using the key structural deformation data obtained from the aforementioned finite element analysis as boundary conditions, it is imported into high-frequency electromagnetic simulation software (e.g., GRASP). The far-field radiation pattern of the antenna is then calculated at the target operational frequency (40 GHz).

**Figure 5:**
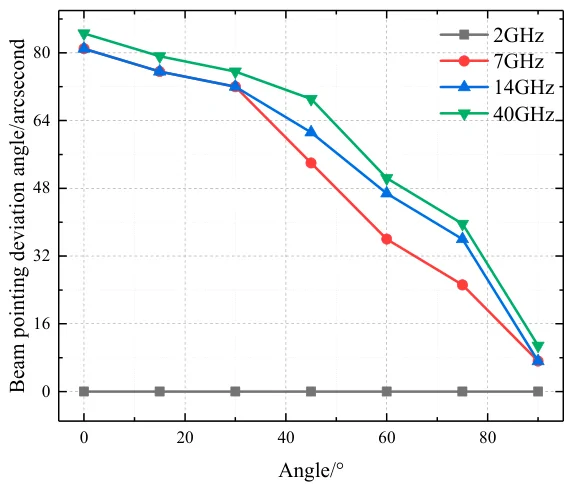
Impact of Combined Effects on Radiation Pattern Pointing.

**Figure 6:**
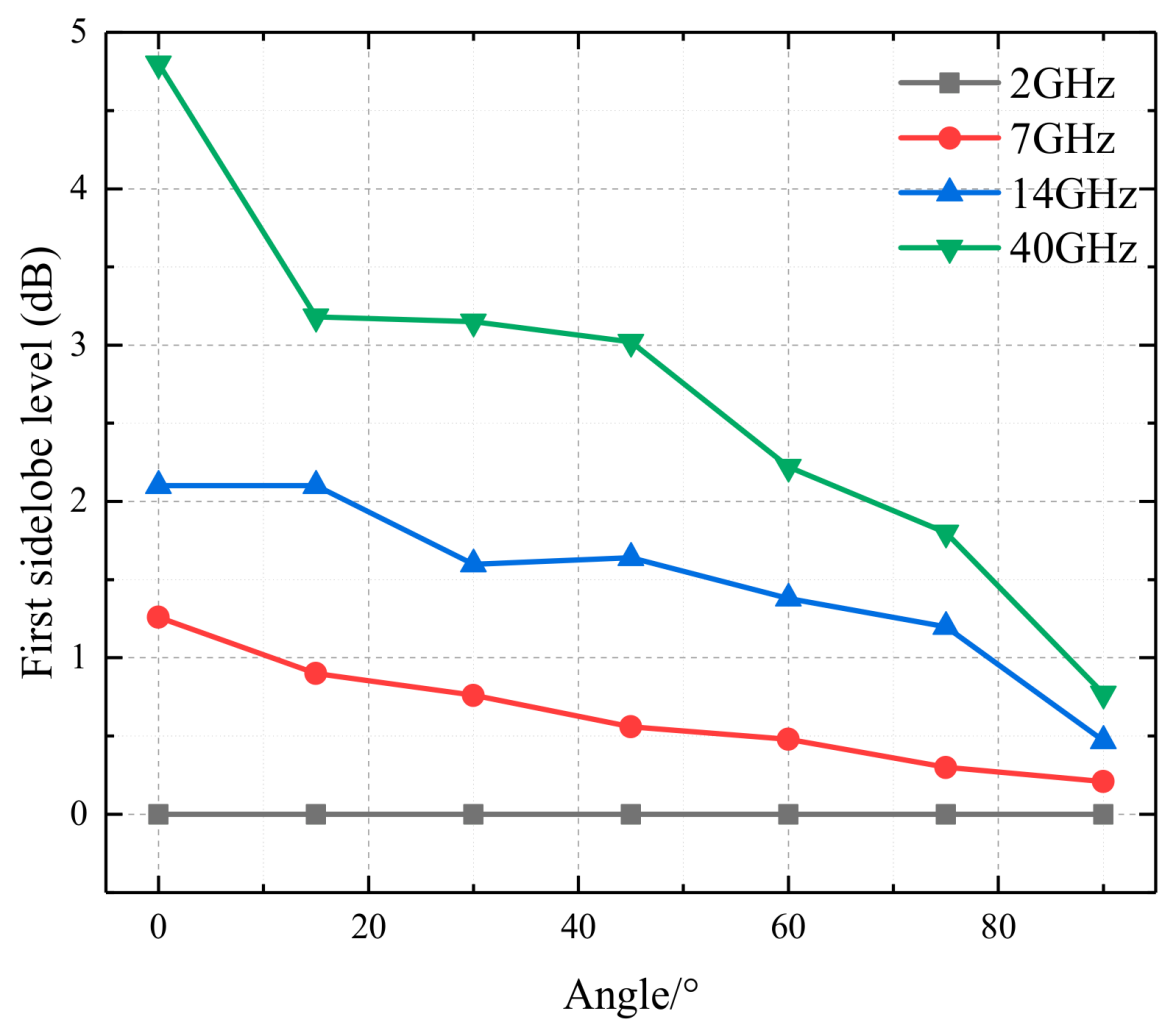
Impact of Combined Effects on the First Sidelobe of the Radiation Pattern.

Considering all deformation factors collectively, As can be seen in Figure 5 and Figure 6, within the elevation angle range of 0° to 60°, the effects of main reflector deformation and feed/sub-reflector displacement on the radiation pattern counteract each other. In contrast, between 60° and 90°, these two deformation factors produce additive effects.

Under the current surface accuracy specifications and gravitational influence, the maximum pointing offset is calculated as 0.024°. A more significant degradation is noted in the first sidelobe level, with the worst-case deterioration approaching 5 dB. Nonetheless, this performance remains acceptable within the operational elevation range below 80°.

#### 2.1.4. Systematic Geometric Errors and Their Integration with Structural Deformation

Systematic geometric errors mainly include main plate non-levelness, axis non-orthogonality, misalignment between the electrical axis and the elevation axis, and encoder zero-offset errors, On the basis of these geometric errors, structural deformation errors need to be systematically integrated into the unified error model through the following approaches:

- **Geometric Mapping**: The displacements of the sub-reflector and feed under the coupled effects of gravity and wind load (see Table 1) are converted into equivalent azimuth and elevation pointing deviations based on the optical path geometry of the antenna. These deviations are incorporated as parametric error terms dependent on both antenna attitude and environmental conditions.
- **Coupling Modeling**: Structural deformation interacts with other systematic errors. For example, gravity-induced deformation alters the installation reference, thereby coupling with encoder zero-offset errors; wind-induced quasi-static structural tilting amplifies the effect of main plate non-levelness errors. Thus, in the modeling framework, structural deformation is not only an independent error source but also a coupling medium that links and influences geometric, gravitational, and environmental errors.

### 2.2. Unified Error Classification and Mathematical Modeling

#### 2.2.1. Systematic Geometric Error

- Main Plate Level Error

When a horizontal inclination angle ϕ exists in the reference plane, the instrument coordinate system $ox_{1}^{\prime }x_{2}^{\prime }x_{3}^{\prime }$ can be regarded as the result of rotating the astronomical reference coordinate system $ox_{1}x_{2}x_{3}$ clockwise around the horizontal axis by this inclination angle. The coordinate transformation relationship between the two can be described by a rotation matrix.

(1) $$T_{ox_{1}x_{2}x_{3}\to ox_{1}^{\prime }x_{2}^{\prime }x_{3}^{\prime }}=\left[\begin{array}{ccc} 1 & 0 & 0\\ 0 & \cos\phi & \sin\phi \\ 0 & -\sin\phi & \cos\phi \end{array}\right]$$

Typically, the horizontal inclination angle ϕ of the reference plate is small, allowing simplification using approximations such as cosϕ≈1,sinϕ≈ϕ. After applying these simplifications, the above expression can be reduced to:

(2) $$\left[\begin{array}{c} x_{1}^{\prime }\\ x_{2}^{\prime }\\ x_{3}^{\prime } \end{array}\right]=\left[\begin{array}{ccc} 1 & 0 & 0\\ 0 & 1 & \phi \\ 0 & -\phi & 1 \end{array}\right]\left[\begin{array}{c} x_{1}\\ x_{2}\\ x_{3} \end{array}\right]$$

Based on the definition of the coordinate system, the relationship between the azimuth/elevation angles and the coordinate system can be derived as follows:

(3) $$\begin{array}{l} \cos\left(A\right)=\frac{x_{1}}{\sqrt{x_{1}^{2}+x_{2}^{2}}},\cos\left(A^{\prime }\right)=\frac{x_{1}^{\prime }}{\sqrt{x_{1}^{\prime 2}+x_{2}^{\prime 2}}}\\ \sin\left(A\right)=\frac{x_{2}}{\sqrt{x_{1}^{2}+x_{2}^{2}}},\sin\left(A^{\prime }\right)=\frac{x_{2}^{\prime }}{\sqrt{x_{1}^{\prime 2}+x_{2}^{\prime 2}}}\\ \tan\left(E\right)=\frac{x_{3}}{\sqrt{x_{1}^{2}+x_{2}^{2}}} \end{array}$$

When the elevation error angle and azimuth error angle are small, the expressions can be approximated as:

(4) $$\begin{array}{l} \Delta A_{1}\approx \sin\left(A^{\prime }-A\right)=\sin A^{\prime }\cos A-\cos A^{\prime }\sin A\\ =\frac{x_{2}^{\prime }}{\sqrt{x_{1}^{\prime 2}+x_{2}^{\prime 2}}}\frac{x_{1}}{\sqrt{x_{1}^{2}+x_{2}^{2}}}-\frac{x_{1}^{\prime }}{\sqrt{x_{1}^{\prime 2}+x_{2}^{\prime 2}}}\frac{x_{2}}{\sqrt{x_{1}^{2}+x_{2}^{2}}}\\ =\frac{\left(x_{2}^{\prime }-x_{2}\right)}{\sqrt{x_{1}^{\prime 2}+x_{2}^{\prime 2}}}\frac{x_{1}}{\sqrt{x_{1}^{2}+x_{2}^{2}}}=\phi \frac{x_{3}}{\sqrt{x_{1}^{\prime 2}+x_{2}^{\prime 2}}}\frac{x_{1}}{\sqrt{x_{1}^{2}+x_{2}^{2}}}\\ \approx \phi \tan\left(E\right)\cos\left(A\right) \end{array}$$

Similarly,

(5) $$\Delta E_{1}=-\phi \sin A$$

Let the azimuth angles (measured from true north) of the directions corresponding to the maximum north–south and east–west level offsets be $A_{M}$, respectively. The resulting azimuth and elevation error angles are given by:

(6) $$\left\{\begin{array}{l} \Delta A_{1}=\phi \tan\left(E\right)\cos\left(A-A_{M}\right)\\ \Delta E_{1}=-\phi \sin\left(A-A_{M}\right) \end{array}\right.$$

b. Non-orthogonality Error between Azimuth and Elevation Axes

**Figure 7:**
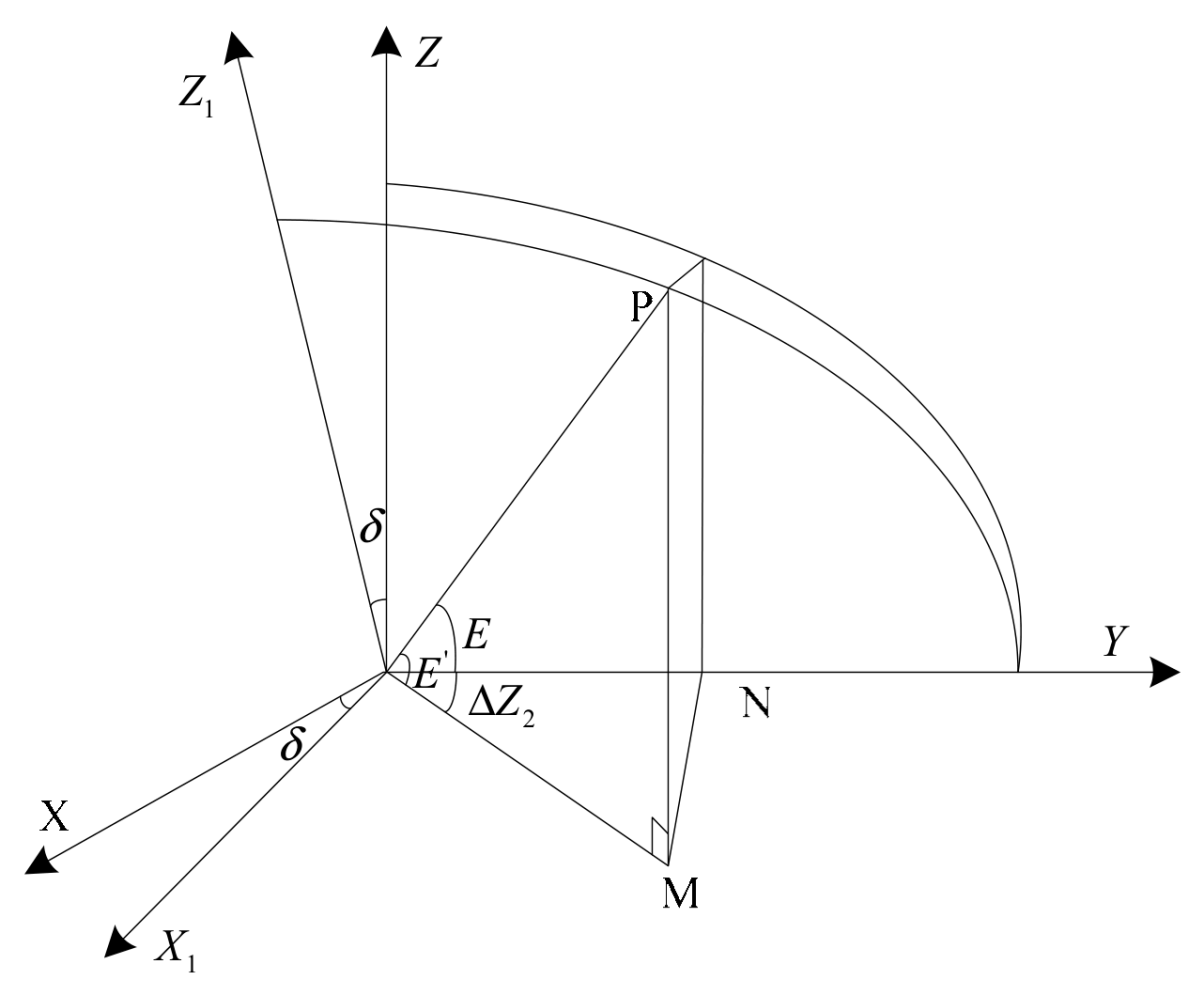
Orthogonality Error between the Elevation and Azimuth Axes.

When no non-orthogonality error exists between the two axes, the azimuth axis aligns with the vertical direction, coinciding with the *Z*-axis, while the elevation axis coincides with the OX-axis. If a non-orthogonality error δ exists between the elevation axis and the azimuth axis, it can be modeled as the coordinate system OXYZ rotating by the angle δ around the OY-axis. This is illustrated in Figure 7 below.

Through coordinate transformation and analysis of geometric relationships, it can be derived that:

When an orthogonality error δ exists between the elevation axis and the azimuth axis, it introduces an azimuth error:

(7) $$\Delta A_{2}=\delta \tan\left(E\right)$$

and an elevation error:

(8) $$\Delta E_{2}=\tan\left(E\right)\left(1-\cos\delta \right)\approx \frac{\delta^{2}}{2}\tan\left(E\right)$$

where δ is the non-orthogonality angle expressed in radians, and the approximation follows from the small-angle expansion $1-\cos\delta \approx \delta^{2}/2$. This notation ensures that only δ is squared, while $\tan\left(E\right)$ remains outside the square.

Since the elevation error is a second-order function of the orthogonality error, it can generally be neglected.

c. Non-orthogonality Error Between the Electrical Axis and the Elevation Axis

**Figure 8:**
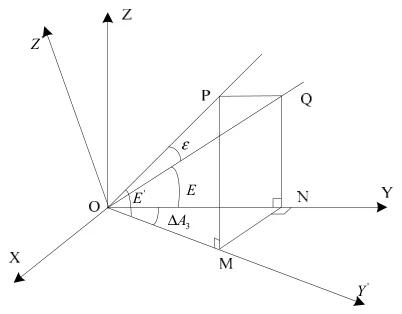
Non-orthogonality Error between the Electrical Axis and the Elevation Axis.

The electrical axis represents the true boresight direction, while the elevation axis corresponds to the encoder’s measurement axis. During measurement, the electrical axis is aligned with the target, and the encoder axis provides the angular readout. A misalignment between these two axes introduces measurement error. In the horizon coordinate system shown in Figure 8 below, the azimuth axis coincides with the *Z*-axis, and the elevation axis coincides with the *X*-axis. When the elevation angle is 0°, the electrical axis coincides with the *Y*-axis. If the electrical axis OQ is offset by an angle ε, then as the elevation axis rotates, the electrical axis no longer moves within the YOZ plane but instead traces a path along MOP.

Based on the geometric relationship illustrated in the figure, the azimuth error induced by the non-orthogonality between the electrical axis and the elevation axis is derived as:

(9) $$\Delta A_{3}=\varepsilon \sec E$$

The elevation error resulting from this misalignment angle is:

(10) $$\Delta E_{3}=\frac{\varepsilon^{2}}{2}\tan E$$

Since the elevation error is a quadratic function of the offset angle ε, it constitutes a second-order small quantity and can generally be neglected.

Encoder zero offset: Constant biases CA (Collimation Azimuth) and CE (elevation).

Gravity-induced deflection: Characterized by a polynomial function of the normalized elevation angle. To improve numerical conditioning and facilitate physical interpretation, the elevation angle is first normalized as $x=El/90^{{}^{\circ}}$, where El is in degrees and $x\in \left[0,1\right]$. The gravity-induced pointing offset is then expressed as:

(11) $$\Delta G(x)=a_{0}+a_{1}\cdot x+a_{2}\cdot x^{2}+\ldots a_{n}\cdot x^{n}$$

where ΔG is in arcseconds, and *x* is dimensionless. The polynomial order *n* is determined based on the structural deformation characteristics (typically *n* = 2 to 4); higher-order terms are omitted to avoid overfitting. The coefficients $a_{0},a_{1},\ldots a_{n}$ are of comparable magnitude (typically a few arcseconds), which reduces the condition number of the fitting matrix and improves numerical stability.

It is important to note that this polynomial is a purely empirical approximation valid only within the elevation range used for fitting (0–90°). Extrapolation beyond this range is not recommended and would require additional structural analysis or measurements. In this work, all pointing corrections are applied within the 0–90° elevation interval, so no extrapolation is involved.

#### 2.2.2. Random Error

**Figure 9:**
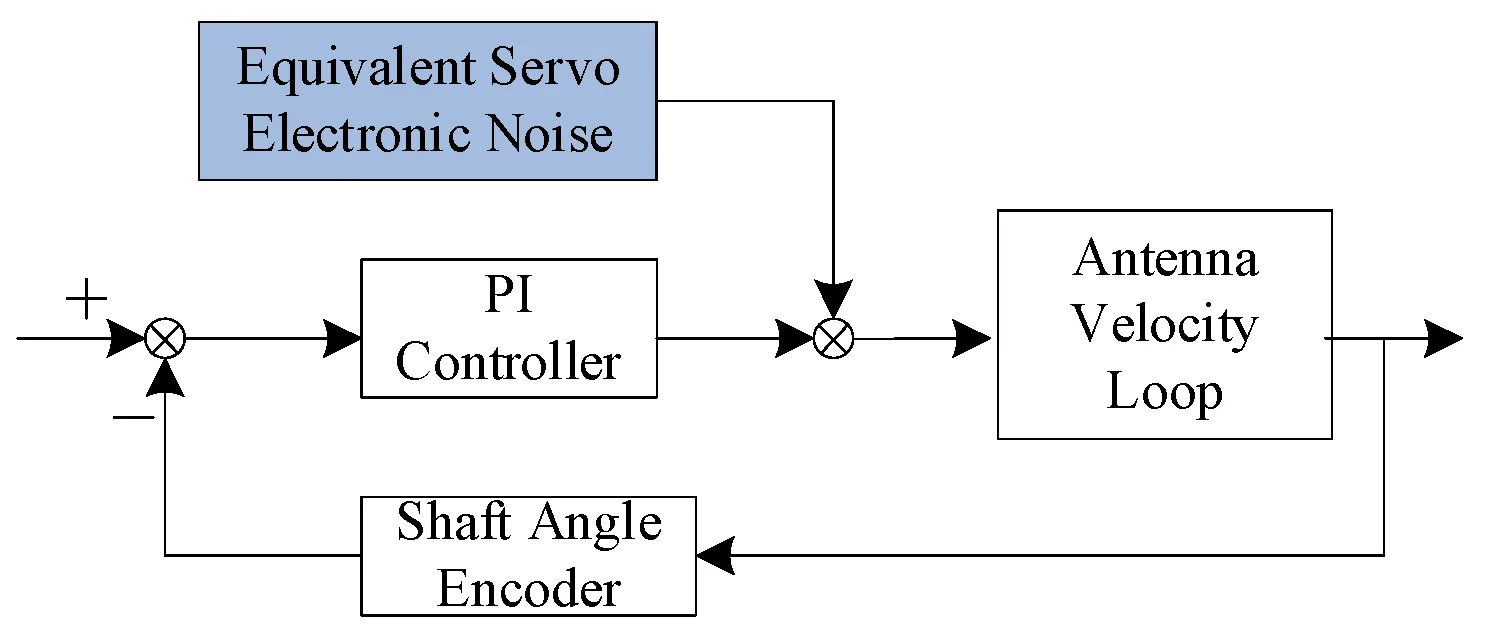
Structure of the Equivalent Servo Electrical Noise Model.

Random Error are primarily driven by servo electrical noise. Servo Electrical Noise originates from the inherent properties, instability, and nonlinearity of circuits and electronic components. In antenna servo systems—where weak control signals drive high-power stages—the primary noise sources reside in components such as control boards and PCCs (Power Control Cabinet). When analyzing the impact of servo electrical noise on pointing error, all electronic noise can be equivalently referred to the input of the antenna’s velocity loop. The structure of this equivalent analytical model is shown in Figure 9 below.

**Figure 10:**
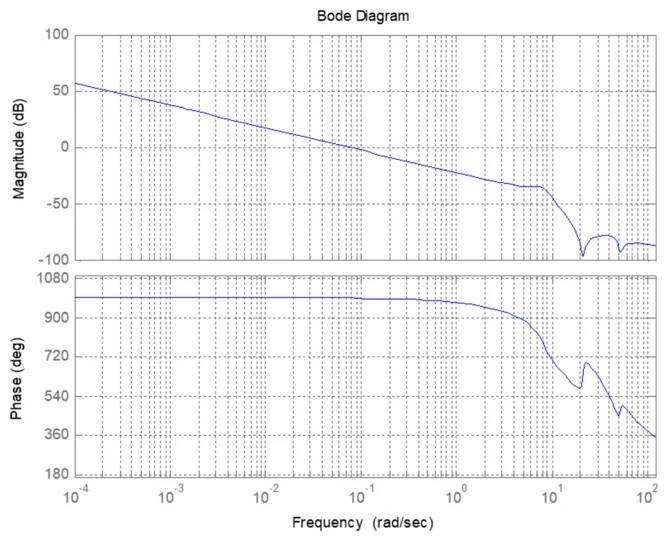
Velocity Loop Bode Plot.

Figure 10 presents the Bode plot of the velocity loop’s amplitude-frequency response. The plot depicts the system’s frequency-dependent gain, with the gain margin and key amplitude values explicitly marked. Analysis of the curve indicates the frequency range over which the loop provides effective response.

#### 2.2.3. Environmental Disturbance Error

Wind-induced deformation: Quasi-static wind pressure induces a peak pointing offset, $\Delta \theta_{wind,RMS}$, taken as the 97.5th percentile from wind-gust statistics. Under the assumption of a zero-mean Gaussian process, the corresponding standard deviation is:

(12) $$\Delta \theta_{wind,RMS}\approx \frac{\Delta \theta_{wind,peak}}{1.96}$$

And the 95% bound is therefore. $\Delta \theta_{wind,95}$. Thus, for wind, the peak value itself equals the 95% limit because of the chosen percentile definition. For other deterministic errors, different conversion factors are used as described in Section 2.3.

#### 2.2.4. Dynamic Tracking Error

The preceding error analysis primarily addresses static or quasi-static conditions. During dynamic scanning, additional errors arise, mainly dynamic lag and drive backlash.

Dynamic Lag: Caused by the finite servo bandwidth, this speed-proportional error is compensated by the servo integrator and feedforward control. Its residual is included in the total budget under “dynamic error.”

Drive Backlash: This refers to lost motion in the mechanical transmission. The project employs a multi-motor active backlash elimination method, yielding a residual of approximately 1–2 arcseconds after compensation.

#### 2.2.5. Analysis of Multi-Error-Source Coupling Mechanisms

Error sources interact through multi-physical coupling. Their combined effect is not a linear sum, governed by three primary mechanisms:

- Geometric-Physical Coupling

Gravitational deformation $\delta_{g}$ is modulated by environmental factors like wind $\delta_{w}$. The total quasi-static deformation is:

(13) $$\delta_{total}=\delta_{g}(El)+\delta_{w}(V,El)+\delta_{cpl}(El,V)$$

where the coupling term $\delta_{cpl}$ captures the nonlinear cross-influence between system and environmental variables.

b. Control-Structure Dynamic Coupling

Servo dynamic errors $\theta_{dyn}$ excite structural vibrations. The resulting dynamic pointing error is a convolution:

(14) $$\Delta \theta_{dyn}(t)=\varepsilon_{dyn}(t)*h(t)$$

where h(t) is the structure’s impulse response. This coupling amplifies deviations during high-bandwidth tracking.

It is important to clarify that the coupling terms introduced in Equations (13) and (14), namely $\delta_{cpl}(EL,\;V)$ and the convolution with *h*(*t*), are qualitatively identified as physical interaction mechanisms. However, under the specific operating conditions of this study (wind speed ≤ 4 m/s, scan rate ≤ 0.1°/s), these coupling effects are quantitatively evaluated as being at least one order of magnitude smaller than the dominant independent error sources (see Table 2). Specifically, the geometric–physical coupling term $\delta_{cpl}(EL,\;V)$ is estimated from the residual of the superposition test: the combined deformation under simultaneous gravity and wind is compared with the linear sum of the individual deformations; the difference (coupling residual) is less than 0.3 arcseconds (RMS@95%) across the entire elevation range. Similarly, the control–structure dynamic coupling is attenuated by the wide bandwidth (>10 Hz) of the servo velocity loop, resulting in a residual dynamic coupling error below 0.2 arcseconds. These estimates are based on the simulation setup described in Section 2.1 and the Bode plot in Figure 10.

Therefore, while the coupled modeling framework is essential for qualitatively understanding the physical interactions among error sources and for guiding the hierarchical compensation strategy, the coupling contributions are negligible in the final error synthesis stage. This justifies the use of the RSS method for error budgeting. In cases where future applications involve higher wind speeds or faster scanning, the coupling terms would need to be re-evaluated and potentially included via covariance terms or Monte Carlo simulation.

c. Coupling in Error Synthesis

To synthesize these different error types, the method mandates the Root Mean Square error at a 95% confidence level (RMS@95%) as the unified metric. For errors characterized by peak-to-peak (P-P) or peak values, conversion follows a Gaussian assumption: RMS ≈ Peak/1.96. The total system pointing error is then synthesized from the RMS values of each independent error source using the Root Sum of Squares (RSS) method:

(15) $$E_{total}=\sqrt{\sum_{i=1}^{n}{\left(E_{i,RMS}\right)}^{2}}$$

In summary, coupling arises from interactions among geometric, physical, and dynamic processes. Its quantitative analysis is essential for accurate error budgeting and targeted compensation. For the representative operating conditions of this antenna (scan rate ≤ 0.1°/s, step-and-settle tracking for radio sources), the current combination of feedforward control and active backlash elimination provides adequate dynamic error compensation. However, under more demanding conditions (e.g., faster scanning, rapidly varying external loads), more advanced adaptive or intelligent control techniques—such as model predictive control, disturbance observer-based control, or neural network-based compensation—would be beneficial for real-time parameter adaptation. These approaches are planned as future work to extend the applicability of the proposed framework to harsher operational scenarios.

### 2.3. Statistical Definition of Pointing Errors

In this study, all error components are quantified using a two-sided 95% confidence bound for a zero-mean Gaussian scalar variable, i.e., 1.96σ, where σ is the standard deviation. This bound is denoted as RMS@95% throughout the paper. For clarity, each entry in the error budget (Table 2) represents this two-sided 95% limit, assuming approximate normality after appropriate processing.

When raw data are obtained as peak or peak-to-peak values, conversion to σ depends on the underlying distribution:

- For a Gaussian random variable, the 95% bound equals 1.96σ. If the “peak” is taken as the 97.5th percentile, then σ = peak/1.96; if a symmetric ±peak-to-peak range is given, then σ = peak-to-peak/3.92.
- For a sinusoidal deterministic error, σ = peak/$\sqrt{2}$.
- For a uniform bounded error, σ = peak/$\sqrt{12}$.

In this work, wind-induced deformation is treated as Gaussian based on statistical analysis of dynamic wind pressure; servo noise and mechanical tolerances are also modelled as Gaussian. Gravity deformation and geometric errors are deterministic functions of elevation; their residuals after TPOINT calibration are characterized by the standard deviation of the fitting residuals, which is then scaled to 1.96σ. All contributions are eventually expressed as 1.96σ, i.e., RMS@95%, to enable consistent budgeting.

Since pointing is a two-dimensional quantity (azimuth and elevation), we report the total pointing error as the root-sum-of-squares (RSS) of the two independent axis errors, each given at its 95% bound. This yields a scalar value that can be directly compared with the half-power beamwidth. For a bivariate normal distribution with equal variances and zero correlation, the exact radial 95% error ellipse would require a different coefficient (≈2.45σ); however, the widely adopted engineering practice for antenna pointing uses the RSS of axis errors as a conservative upper bound, which we adopt here and state explicitly.

## 3. Error Budget

Based on the completed multi-error-source modeling and unified characterization, this chapter describes how to systematically synthesize the dispersed error terms to predict the overall antenna pointing accuracy under the most severe operating conditions. This process, termed “error budgeting,” bridges design specifications with engineering implementation.

The core of budgeting involves decomposing complex system errors into several mutually independent or weakly correlated components. Following the established classification framework, all error components are quantified using the root mean square value at a 95% confidence level (RMS@95%) as the unified metric. For the four identified major error categories—servo subsystem error, antenna structural subsystem error, environmental disturbance error, and dynamic tracking error—their residual magnitudes after applying corresponding correction or suppression measures are individually assessed, as summarized in the table below:

For gravity deformation, the residual after GCE compensation is estimated from the standard deviation of the fitting residuals of the polynomial (Equation (11)) over the elevation range 0–90°, expressed as a 95% bound (1.96σ).

### 3.1. Sensitivity Analysis

To assess the robustness of the error budget under parameter uncertainties, we perform a simplified sensitivity analysis by perturbing the key error sources within their expected tolerance ranges. The following parameters are varied independently:

- Gravity deformation model coefficients ($a_{0},a_{1},\ldots a_{n}$): ±5% variation in each coefficient, based on typical fitting uncertainty of finite-element simulations.
- Wind load deformation: ±5% variation in the peak deflection, accounting for uncertainty in wind-gust statistics.
- Servo electrical noise: ±3% variation, representing component tolerances and aging.
- Mechanical assembly errors (e.g., axis non-orthogonality): ±5% variation in residual values after calibration.

Each parameter is perturbed individually while keeping others at their nominal values. The resulting change in the total system pointing error (RSS@95%) is summarized in Table 3 below:

The results indicate that the total pointing error remains within 8.6″–9.0″ under these perturbations, which is still well below the 1/10 HPBW requirement. The wind load deformation is identified as the most sensitive parameter, consistent with its dominant role in the error budget. This sensitivity analysis confirms that the proposed error budget is robust against reasonable parameter uncertainties.

It should be noted that long-term effects such as structural aging, thermal fatigue, and unmodeled disturbances are not included in this analysis. These factors are expected to be slowly varying and can be periodically recalibrated using the TPOINT model (Level 2 compensation) in routine operations. A full long-term robustness assessment is beyond the scope of this work and is planned for future study.

### 3.2. Analysis Process

The error budgeting process, following the unified RMS@95% metric and RSS synthesis method, yields a theoretical total system pointing error of 8.64 arcseconds. This meets the critical design requirement of being ≤1/10 HPBW (Half-Power Beam Width) for 40 GHz operations.

A key finding is the clear identification of the primary error source: Environmental Disturbance Error (7.71 arcseconds), dominated by wind load deformation, constitutes the major limiting factor for further accuracy improvement. This highlights the critical challenge of mitigating environmental effects in high-precision systems.

The validity of the RSS synthesis relies on the assumption that the error components are mutually independent or only weakly correlated. To verify this, we have quantitatively assessed the cross-correlations among the major error sources using the simulation data from Section 2.1. The estimated covariance terms $2\sum_{i<j}Cov(e_{i},e_{j})$ are found to be less than 0.5% of the total RSS value (approximately 0.04 arcseconds in squared sum), which is well below the resolution limit of the budget. Hence, the independent-error assumption is practically justified for this specific antenna system under the stated operating conditions. The coupling mechanisms discussed in Section 2.2 are physically present but do not introduce significant cross-correlations after the hierarchical compensation measures are applied.

## 4. Hierarchical Coordinated Compensation Strategy

This paper proposes a “Hierarchical Collaborative Compensation Strategy”, decomposing the compensation task into three logical levels: Fundamental Suppression, Core Calibration, and Active Enhancement. This creates a complete chain from static to dynamic and hardware to software.

**Level 1: Precision Mechanical Adjustment.** This hardware-based level minimizes foundational errors during installation to establish a high-precision baseline. Key steps include: leveling the main base plate using laser trackers to control azimuth axis tilt; calibrating the azimuth-elevation axis orthogonality and electrical axis collimation; and setting encoder mechanical zero points. This reduces constant systematic errors, easing the burden on subsequent software compensation.

**Level 2: Parametric Model Calibration.** This software-based core level compensates for repeatable systematic errors using a parametric model (TPOINT) fitted with compact radio-source pointing observations. Its key innovation is establishing an explicit mapping between physical error sources (e.g., base non-levelness) and model parameters (e.g., IA, IE). This transforms calibration from “black-box fitting” into “transparent physical diagnostics,” improving debugging. Parameters are solved via least-squares fitting and embedded into the control system for real-time pointing correction. The corresponding mapping between physical error sources and TPOINT model parameters is summarized in Table 4.

**Level 3: Active Servo Suppression.** As the final layer, this stage focuses on the real-time suppression of residual random and dynamic errors. Key measures include multi-motor active backlash elimination to cancel gear transmission lost motion, feedforward control to compensate for dominant dynamic lag, and a high-stiffness, wide-bandwidth velocity loop to improve disturbance rejection. This ensures the antenna maintains accuracy during dynamic tracking and under environmental disturbances.

The three-level strategy operates collaboratively, forming a closed loop of mechanical benchmarking, model-based correction, and control safeguarding. Fundamental suppression provides a favorable baseline for model calibration; the parametric model resolves most systematic deviations; and active suppression handles remaining non-repeatable disturbances. This architecture clarifies the role and interface of each level and is key to translating theoretical accuracy into engineering reality.

### Practical Implementation Considerations

To ensure the long-term effectiveness of the hierarchical compensation strategy, several practical measures have been implemented. Mechanical alignment using precision laser trackers establishes a one-time baseline during installation; the antenna structure is designed with sufficient rigidity and thermal compensation to minimize long-term drift of critical alignment parameters. The TPOINT model is recalibrated at regular intervals (e.g., every 3–6 months) to capture slow variations due to structural settling, thermal effects, or component wear. Active servo suppression provides real-time adaptive compensation for short-term environmental disturbances. In addition, the antenna is equipped with health monitoring devices (e.g., inclinometers, temperature sensors) to detect anomalous drifts and trigger recalibration when necessary. These measures ensure that the proposed strategy remains a maintainable engineering solution throughout the antenna’s operational lifetime.

## 5. Experimental Validation with Radio Source Tracking

To validate the effectiveness of the proposed three-level hierarchical compensation strategy, a series of experiments were conducted on the 18-m antenna. All experiments were carried out under the following conditions: X-band for pointing error measurements, wind speed ≤ 4 m/s, and stable temperature. The validation consists of two complementary parts: static pointing accuracy verification (Figure 1 and Figure 2) and dynamic tracking performance verification (Figure 3 and Figure 4).

### 5.1. Static Pointing Accuracy Verification

The experiments followed the standard cross-scanning procedure commonly used in radio telescope pointing calibration: the antenna scans across a calibration radio source in both azimuth and elevation directions, and the received signal power is recorded as a function of the pointing offset. The measured pointing errors are then obtained by fitting the scan profiles with a Gaussian model.

Step 1—Establishing the baseline (Level 1: Mechanical Alignment): Before any pointing measurements, the mechanical alignment was performed using precision laser trackers, including main base plate leveling, axis orthogonality calibration, electrical axis collimation, and encoder zero-point setting. This established the baseline for all subsequent experiments.

Step 2—TPOINT model calibration and static pointing validation (Level 2): Multiple compact radio sources distributed across the sky were selected as calibration targets to cover a wide range of elevation angles. Open-loop pointing error measurements were performed first to obtain the raw error data. The TPOINT model parameters (AN/NPAE/CA/IA/IE/GCE, etc.) were then fitted using these data. After embedding the model into the control system, independent test sources (different from those used for fitting) were re-scanned to validate the residual pointing errors after TPOINT calibration.

**Figure 11:**
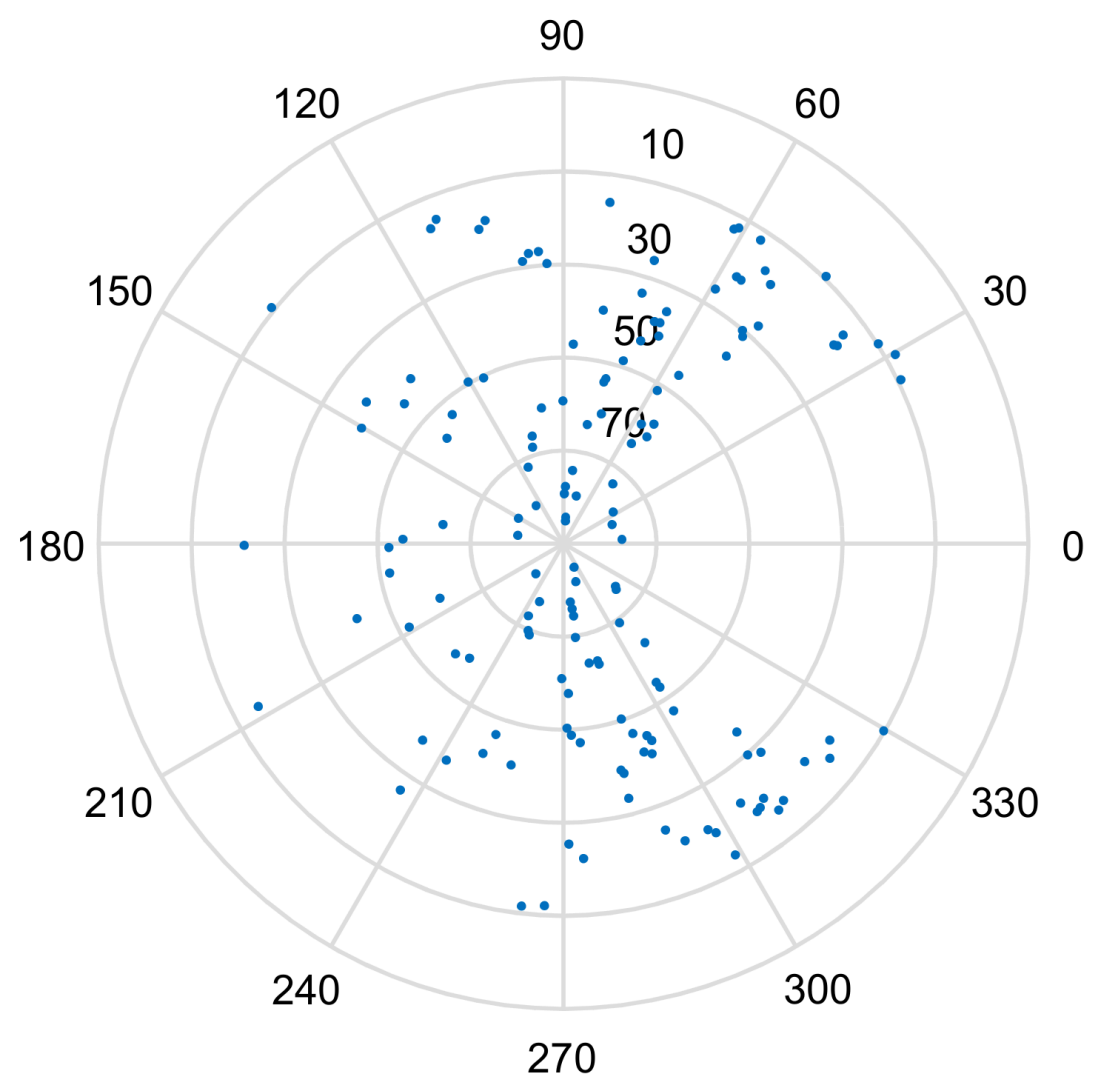
Distribution of pointing error measurement points at X-band.

**Figure 12:**
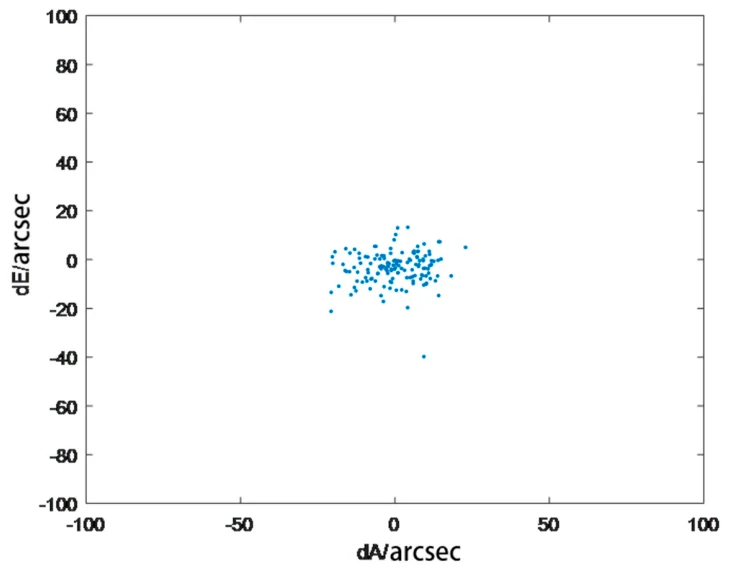
Distribution of residual pointing errors at X-band after TPOINT calibration.

Figure 11 shows the distribution of visible calibration radio sources in the sky (azimuth–elevation two-dimensional coverage) at the time of the experiments; the sources uniformly cover a wide range from low to high elevation angles, ensuring effective model fitting across the entire operational elevation range. Figure 12 presents the distribution of residual pointing errors after TPOINT calibration.

The statistical results are summarized in Table 5.

The results demonstrate that the systematic errors (dominated by gravity deformation and axis misalignments) are effectively compensated by TPOINT calibration, reducing the pointing error to approximately 7.9″, which is well within the 1/10 HPBW requirement at 40 GHz. This confirms the effectiveness of Level 1 and Level 2.

### 5.2. Dynamic Tracking Performance Verification

To validate the effectiveness of Level 3 (active servo suppression) in dynamic tracking, two representative scenarios were tested. In both scenarios, the antenna was commanded to move at maximum speed from its initial position to the target position. The closed-loop settling time is defined as the time required for the tracking error to converge to within ±0.005° after the antenna velocity reaches 1°/s. This definition ensures a consistent and objective measure of dynamic response performance across different test conditions.

**Figure 13:**
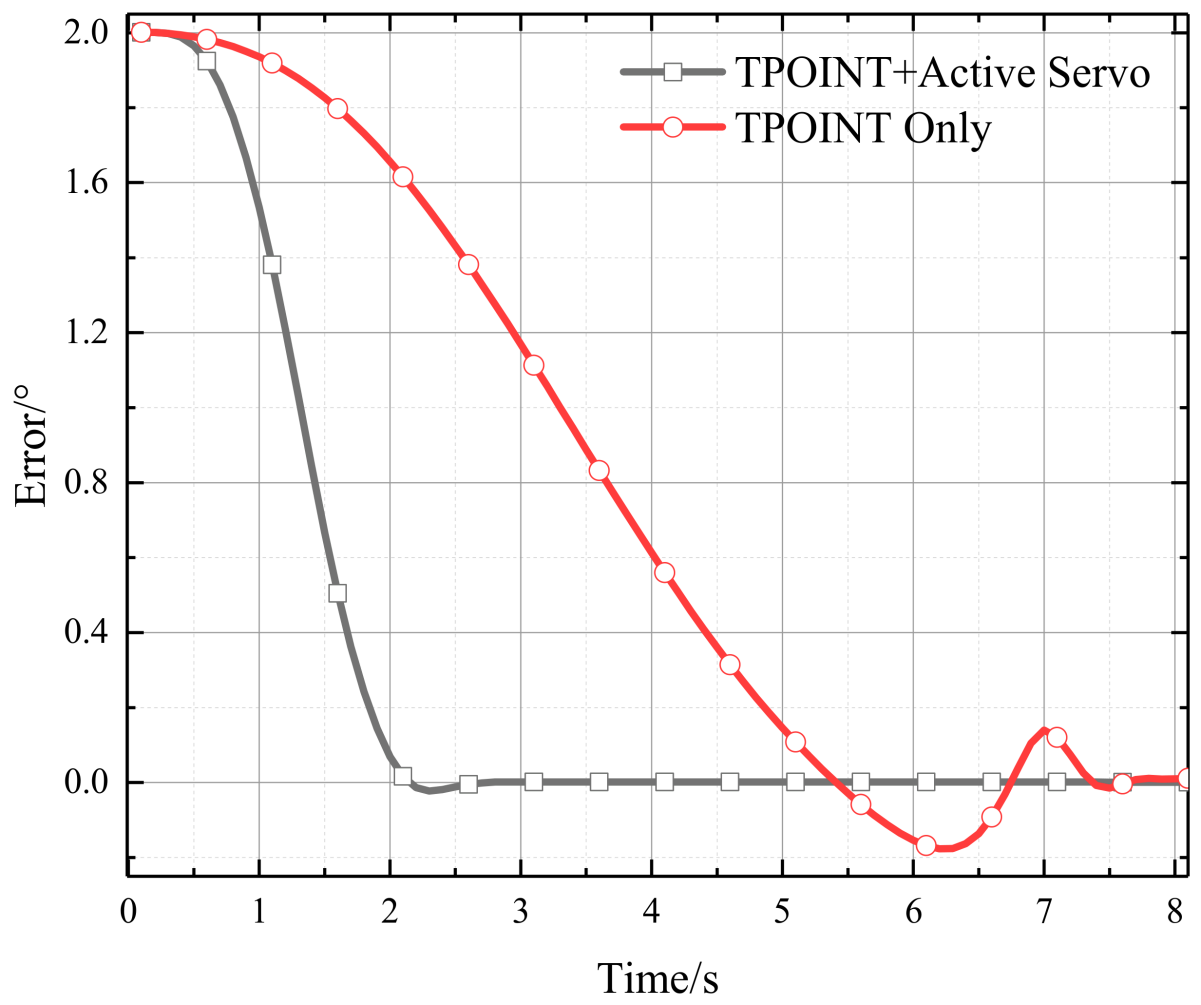
Closed-loop tracking error curves for short-range fast acquisition scenario.

Scenario A—Short-range fast acquisition (Figure 13): The antenna was commanded to move from 0° to 2° at maximum speed. This scenario simulates small-angle target switching or fine-adjustment maneuvers commonly encountered during radio source tracking. The closed-loop tracking error curves were compared between two configurations: TPOINT calibration only (Levels 1 & 2) and TPOINT combined with active servo suppression (Levels 1, 2 & 3). The experimental results are summarized in Table 6.

**Figure 14:**
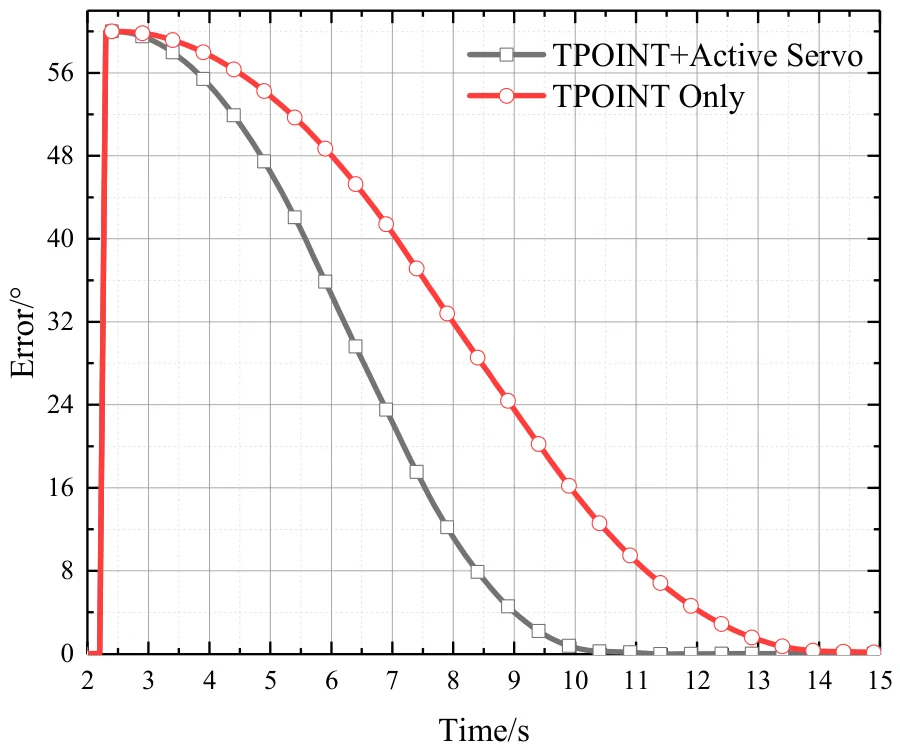
Closed-loop tracking error curves for long-distance large-angle slew scenario.

Scenario B—Long-distance large-angle slew (Figure 14): The antenna was commanded to move from 0° to 60° at maximum speed. This scenario simulates large-span source switching or emergency repositioning, where gravitational deformation and nonlinear friction effects are most pronounced. The closed-loop tracking error curves were compared under the same two configurations. The experimental results are summarized in Table 7.

The above results demonstrate that active servo suppression (feedforward control + multi-motor active backlash elimination) significantly improves dynamic response performance in both scenarios:

In the short-range fast acquisition scenario (0° to 2°), the settling time is reduced from 2.986 s to 1.478 s, an improvement of approximately 50.5%. In the long-distance large-angle slew scenario (0° to 60°), the settling time is reduced from 3.356 s to 1.789 s, an improvement of approximately 46.7%.

The settling time is reduced by approximately 50% in both scenarios, indicating that active servo suppression provides consistent and significant performance improvement across different motion amplitudes. In comparison, the settling time for the large-angle scenario (3.356 s) is longer than that for the small-angle scenario (2.986 s) because gravitational torque variations and nonlinear friction effects are more pronounced during large-angle motion, imposing higher demands on the dynamic response of the servo system. With active servo suppression enabled, the settling times in both scenarios are reduced to approximately 1.5–1.8 s, demonstrating that feedforward control effectively compensates for most of the dynamic lag and nonlinear disturbances.

These results validate the critical role of Level 3 in the three-level strategy: TPOINT calibration (Level 2) addresses static pointing accuracy, while active servo suppression (Level 3) ensures rapid convergence and stable tracking under dynamic conditions. The two are complementary, and both are indispensable for achieving the overall pointing performance required at 40 GHz.

## 6. Conclusions

This paper presents a high-precision pointing method for high-frequency VLBI antennas based on multi-error-source coupled modeling and hierarchical compensation. The system achieves a pointing accuracy of 8.64 arcseconds under moderate conditions (wind ≤ 4 m/s, scan rate ≤ 0.1°/s), meeting the ≤1/10 HPBW requirement at 40 GHz. The main innovation lies in a three-level compensation framework—mechanical adjustment, TPOINT model calibration, and active servo suppression—which incorporates explicit mapping between physical error sources and model parameters for transparent correction. Future work may extend the method to harsher conditions via adaptive compensation and intelligent calibration.

To validate the proposed method, actual radio source tracking experiments were conducted on the 18-m antenna. The experimental results demonstrate that after applying the three-level hierarchical compensation strategy, the residual pointing error is reduced to approximately 7.9″ (RMS@95%), which agrees well with the theoretical budget of 8.64″. This confirms the practical effectiveness of the proposed framework in realistic operational scenarios.

This study assumes wind speeds ≤ 4 m/s and scan rates ≤ 0.1°/s. Performance may degrade under higher winds or faster scanning due to increased dynamic errors. The method is thus suited for moderate environments, and more extreme conditions would require enhanced adaptive compensation. In addition, the sensitivity analysis confirms that the error budget remains robust within the studied parameter tolerances; however, long-term effects such as structural aging and thermal fatigue are not yet addressed and will be investigated in future work through periodic recalibration and in situ monitoring.

## Figures and Tables

**Table 1:** Variation in Sub-reflector and Feed Displacement with Elevation Angle.

Elevation Angle	Sub-Reflector Displacement (Gravity Direction)	Feed Displacement (Gravity Direction)	Unit
0	4.997	1.3664	mm
10	4.989849	1.450507	mm
20	4.831084	1.490542	mm
30	4.525529	1.485287	mm
40	4.082468	1.434903	mm
50	3.515363	1.340919	mm
60	2.841446	1.206193	mm
70	2.081193	1.034817	mm
80	1.257704	0.831998	mm
90	0.396	0.6039	mm

**Table 2:** Residual Error Values.

Error Type	Corrected Residual (Arcseconds, RMS@95%)	Remarks/Mapping
**A. Servo Subsystem Error**	1.90	Combined electrical noise, resolution, nonlinearity
**B. Antenna Structural Subsystem Error**	3.07	RSS result of sub-items
B1: Azimuth Axis Non-verticality	(Included)	Residual after IA (Index error of Azimuth axis), IE (Index error of Elevation axis) compensation
B2: Azimuth-Elevation Axis Non-orthogonality	(Included)	Residual after NPAE (Non-Perpendicularity Azimuth-Elevation axes error) compensation
B3: Random Mechanical Errors	(Included)	Bearing runout, assembly tolerances, etc.
B4: Gravity Deformation Residual	(Included)	Residual after GCE(El) (Gravity Correction for Elevation) compensation
**C. Environmental Disturbance Error**	7.71	RSS result of sub-items
C1: Wind Load Deformation (≤4 m/s)	7.60	Primary error source
C2: Thermal Gradient Deformation Residual	1.27	
**D. Dynamic Tracking Error**	1.50	0.1°/s scanning, lag & backlash residual
**Total System Pointing Error (RSS Synthesis)**	**8.64**	Theoretical budget value

**Table 3:** Total system pointing error.

Perturbed Parameter	Variation Range	Change in Total Pointing Error
Gravity deformation model coefficients	±5%	+0.14″/−0.11″
Wind load deformation	±5%	+0.36″/−0.29″
Servo electrical noise	±3%	+0.07″/−0.06″
Mechanical assembly errors	±5%	+0.04″/−0.03″

**Table 4:** TPOINT Model Parameter Mapping.

Physical Error Source	TPOINT Parameter	Physical Meaning of Parameter	Treatment in This Model
Main Plate Non-levelness (Leveling Residual)	AN (Azimuth axis misalignment, North–South) (or AW (Azimuth axis misalignment, East–West))	Tilt of the azimuth axis	Compensated via AN; residual included in budget.
Non-orthogonality between Azimuth and Elevation Axes	NPAE	Non-perpendicular angle error	Compensated via NPAE; residual included in budget.
Non-orthogonality between Electrical and Elevation Axes	CA	Azimuth collimation error	Compensated via CA; residual included in budget.
Encoder Zero-point Error	IA, IE	Encoder zero offset	Compensated via IA, IE; residual included in budget.
Gravity Deformation (Main Reflector, Support Legs, etc.)	ΔG	Gravity sag effect	The ΔG term uses a normalized elevation polynomial (Equation (11)) to ensure numerical stability during least-squares fitting.
Wind/Thermal Deformation, Dynamic Errors	(Not Mapped)	Non-repeatable or random errors	Not included in the TPOINT model.

**Table 5:** Static Pointing Error Statistics Before and After TPOINT Calibration.

Metric	Before Compensation	After TPOINT Calibration
Pointing error (RMS@95%) OF AZ	~25″–35″	5.6″
Pointing error (RMS@95%) OF EL	~25″–35″	5.6″
Pointing error (RMS@95%)	~35″–50.5″	7.9″

**Table 6:** Dynamic Tracking Performance for Short-Range Fast Acquisition (0° to 2° Step).

Metric	TPOINT Only	TPOINT + Active Servo
Settling time (to ±0.005°, from 1°/s)	2.986	1.478

**Table 7:** Dynamic Tracking Performance for Long-Distance Large-Angle Slew (0° to 60° Step).

Metric	TPOINT Only	TPOINT + Active Servo
Settling time (to ±0.005°, from 1°/s)	3.356	1.789

## Data Availability

The data presented in this study are available upon request from the corresponding author.
